# Outcomes among buprenorphine-naloxone primary care patients after Hurricane Sandy

**DOI:** 10.1186/1940-0640-9-3

**Published:** 2014-01-27

**Authors:** Babak Tofighi, Ellie Grossman, Arthur R Williams, Rana Biary, John Rotrosen, Joshua D Lee

**Affiliations:** 1 Department of Population Health, New York University School of Medicine, 227 E.30th St. 7th floor, New York, NY 10016, USA; 2 Division of General Internal Medicine, New York University School of Medicine, 462 First Avenue, Medicine Clinic, New York, NY 10016, USA; 3 Department of Psychiatry, New York University School of Medicine, One Park Avenue, New York, NY 10016, USA; 4 Department of Emergency Medicine, New York University School of Medicine, New York, USA

## Abstract

**Background:**

The extent of damage in New York City following Hurricane Sandy in October 2012 was unprecedented. Bellevue Hospital Center (BHC), a tertiary public hospital, was evacuated and temporarily closed as a result of hurricane-related damages. BHC’s large primary care office-based buprenorphine clinic was relocated to an affiliate public hospital for three weeks. The extent of environmental damage and ensuing service disruption effects on rates of illicit drug, tobacco, and alcohol misuse, buprenorphine medication supply disruptions, or direct resource losses among office-based buprenorphine patients is to date unknown.

**Methods:**

A quantitative and qualitative semi-structured survey was administered to patients in BHC’s primary care buprenorphine program starting one month after the hurricane. Survey domains included: housing and employment disruptions; social and economic support; treatment outcomes (buprenorphine adherence and ability to get care), and tobacco, alcohol, and drug use. Open-ended questions probed general patient experiences related to the storm, coping strategies, and associated disruptions.

**Results:**

There were 132 patients enrolled in the clinic at the time of the storm; of those, 91 patients were recruited to the survey, and 89 completed (98% of those invited). Illicit opioid misuse was rare, with 7 respondents reporting increased heroin or illicit prescription opioid use following Sandy. Roughly half of respondents reported disruption of their buprenorphine-naloxone medication supply post-event, and self-lowering of daily doses to prolong supply was common. Additional buprenorphine was obtained through unscheduled telephone or written refills from relocated Bellevue providers, informally from friends and family, and, more rarely, from drug dealers.

**Conclusions:**

The findings highlight the relative adaptability of public sector office-based buprenorphine treatment during and after a significant natural disaster. Only minimal increases in self-reported substance use were reported despite many disruptions to regular buprenorphine supplies and previous daily doses. Informal supplies of substitute buprenorphine from family and friends was common. Remote telephone refill support and a temporary back-up location that provided written prescription refills and medication dispensing for uninsured patients enabled some patients to maintain an adequate medication supply. Such adaptive strategies to ensure medication maintenance continuity pre/post natural disasters likely minimize poor treatment outcomes.

## Background

Hurricane Sandy struck the Northeastern United States on Monday, October 29, 2012. It was the largest and second costliest hurricane in United States’ history that resulted in 117 deaths and approximately $50 to 65 billion in damages [1,2]. Bellevue Hospital Center (BHC), a public sector tertiary referral center in the New York City Health & Hospitals Corporation (HHC) network, was closed after flood tides damaged the hospital’s basement and vital infrastructure, leaving inpatient services closed until February 2013. Outpatient facilities at BHC were closed pre-emptively one day prior to Sandy, and did not re-open for three weeks post-event. During this outpatient closure, ambulatory medical, mental health, and pharmacy services were relocated to Metropolitan Hospital, an affiliate HHC public hospital seventy blocks (3.5 miles) north in Manhattan.

The disaster led to the suspension of office-based operations of BHC’s primary care buprenorphine practice, along with a variety of citywide hospital, outpatient clinic, and pharmacy closures. Since 2006, BHC has operated a large primary care office-based buprenorphine practice serving predominantly Medicaid and uninsured patients [3,4]. Care is provided by buprenorphine-waivered internal medicine physicians. At the time of Hurricane Sandy, 132 adults were actively in the clinic.

On the second day after the Hurricane (Wednesday, October 31st), while the inpatient units of the hospital were being evacuated, Bellevue ambulatory care staff downloaded patient schedule information for targeted groups of ‘high-risk’ patients. These patients included those on blood thinners (e.g. Coumadin) and those on chronic opioid maintenance (e.g. the buprenorphine clinic). Using this list of patient phone numbers, the buprenorphine program director attempted to make contact with patients in order of appointments scheduled, starting with those who had already had appointments cancelled that week. Since entrance to the hospital was barred for non-staff, arrangements were made to mail prescriptions for those who had Medicaid or other insurance; for others, there were initially a few ad-hoc patient-doctor meetings immediately outside the hospital entrance to provide prescriptions and directions to other public hospitals. Within the first week after the hurricane, arrangements were made to house a Bellevue-staffed outpatient walk-in clinic for Bellevue patients at an alternate public hospital location; BHC buprenorphine patients who needed to be seen were scheduled for appointments at this location on days/times that a buprenorphine-certified physician was present. Outside of these phone calls from the clinic director, some patients contacted the buprenorphine clinic physicians via phone numbers ‘saved’ from previous between-visit communications or via online searches for email addresses. BHC’s outpatient facilities, including the buprenorphine program, were reopened three weeks after the hurricane (November 19, 2012). Inpatient hospital services did not reopen until mid-February 2013.

### Disasters and its impact on substance use

As providers and health services researchers, we were concerned with direct and indirect effects of the hurricane itself (i.e. flooding) and transit, employment, and health system disruptions on our patients and their opioid treatment outcomes. Previous research indicates that the relationship between disaster exposure and substance use patterns is complex. Psychiatric co-morbidities, cultural factors, and disaster-related exposures have been associated with diverse post-event substance use patterns. For instance, while post-traumatic stress disorder and resource loss have been associated with increased substance misuse post-disaster, cultural and social factors were suggested as potential deterrents to alcohol consumption following the 1995 Hanshin earthquake in Japan [5-9]. Furthermore, acute reductions in the availability of illicit substances and disruptions in employment and subsequent economic constraints have been attributed with an increased demand for drug and alcohol treatment [10,11].

Studies on post-disaster substance use among patients enrolled in addiction treatment programs have been limited. Participants in New York City completing alcohol detoxification prior to the September 11^th^ attacks had higher rates of relapse post-event compared to the period preceding the attacks [12]. Among opioid dependent patients enrolled in methadone treatment programs (MTP) and intensive outpatient programs (IOP), higher rates of illicit substance use and positive urine samples were also reported following the attacks [13]. While reports of inaccessible or closed MTP’s may have led to increases in heroin and prescription misuse following Sandy [14], substance use patterns among office-based buprenorphine patients have yet to be described.

The intention of this exploratory study was to determine self-reported illicit opioid use (other than illicitly-obtained buprenorphine); self-reported tobacco, alcohol, and drug misuse; coping strategies following buprenorphine supply disruption, and resource loss among opioid-dependent patients enrolled in BHC’s office-based buprenorphine clinic immediately following Hurricane Sandy.

## Methods

### Population and recruitment

The population of interest consisted of opioid-dependent patients enrolled in the BHC primary care office-based buprenorphine program at the time of the event (N = 132). Eligible participants were adult opioid dependent patients with an active buprenorphine prescription from a BHC primary care provider on October 30, 2012. Consecutive sampling occurred between November 27th, 2012, and February 4^th^, 2013. The New York University School of Medicine Institutional Review Board approved the study protocol.

### Survey instrument and analysis

A 52-item administered survey instrument was developed and refined by the research team following a review of the disaster, addiction, and post-traumatic stress literature. The survey assessed self-reported pre/post estimates of drug use, treatment adherence, health behaviors, and environmental effects of the hurricane using discreet and open-ended items. Baseline demographic characteristics (gender, age, race/ethnicity, housing, employment and financial status) anchored items querying changes in housing, employment, income and social and government support post-event. Alcohol and drug (tobacco, opioids, stimulants, benzodiazepines, and marijuana) use items were adapted from the Addiction Severity Index [15]. Participants were asked to estimate their use of tobacco, alcohol, and drugs during the 12 weeks prior to the hurricane and 4 weeks post-event. Treatment outcomes (buprenorphine supply, coping strategies following treatment or medication supply disruption, overall access to care) prior to and following the hurricane were specific to the office-based practice and buprenorphine maintenance. Open-ended questions probed general peri-disaster experiences, drug and alcohol use, treatment disruptions, and coping strategies.

### Data collection and analysis

Patients were approached in the clinic waiting area or contacted by phone by the research team to participate in the study. They were informed that all responses would be kept confidential and that participation would have no impact on their regular medical care. Surveys were administered by the research team by phone or in person in a private clinic room; two of the interviewers were also buprenorphine clinic physicians, who attempted to minimize coercion and response bias by explaining that participation in the study and survey responses would not be shared with other buprenorphine providers or affect their clinical care in any manner. Interviews lasted approximately 15 minutes and were conducted in a private clinic space or by phone. Patients not surveyed during a clinic session were contacted by telephone. Five phone attempts were made to contact each patient as necessary. Interviews were recorded in writing and de-identified survey responses were entered into an electronic database. Analysis was descriptive (counts, proportions, rates of self-reported outcomes of interest). Open-ended responses were coded using thematic analysis for emergent themes.

## Results

### Recruitment and demographics

Of the 132 eligible patients enrolled in the clinic at the time of Sandy, a total of 91 patients were invited to participate in the study. No patients refused to participate; however, 2 of 91 participants refused to complete the entire survey. Most participants were surveyed within 2 months of the hurricane (71%), while the remaining 29% were surveyed 3–4 months post-event. The 41 patients not interviewed appeared to be a combination of: a) not in clinic and not reachable by phone (drop outs), or, b) active in clinic post-event but missed by the interviewers and not reachable by phone (often due to visit intervals being longer than 4–8 weeks). The study sample was predominately male (82%), White non-Hispanic (42%). All were active buprenorphine maintenance patients with a mean time in treatment of 28 months and an average buprenorphine dose of 20 mg/day (Table 1).

**Table 1:** Study sample demographic characteristics

	**Study sample, n = 91 n (%)**	**Non-responders, n = 41 n (%)**
Male	73 (82)	36 (86)
*Insurance*		
Medicaid	52 (57)	27 (66)
Uninsured	23 (25)	8 (20)
Medicare	10 (11)	0 (0)
Private	9 (10)	6 (15)
*Race/Ethnicity*		
Caucasian	38 (42)	12 (29)
African-American	34 (37)	17 (42)
Hispanic	18 (20)	12 (29)
Asian	1 (1)	0 (0)
*Buprenorphine treatment history*		
Median buprenorphine dose, (range)	20 mg/day (1.5, 24)	16 mg/day (1, 24)
Mean time in treatment (months), (range)	28 (1, 71)	32 (1, 75)

### Heroin and prescription opioid misuse

Few self-reported increased or new-onset heroin (7%) or prescription drug use (1%) following the hurricane; only four participants reported illicit opioid misuse in the three months preceding the hurricane (4%). Two of the six heroin users and the one prescription opioid user reported illicit opioid misuse after running out of buprenorphine. Two participants reported using heroin regularly with no change pre/post; one participant reported decreased heroin use post-event. When asked about cost, purity, and access to heroin, two participants reported increased police presence surrounding street-level drug dealing, increased cost, and decreased purity following Sandy.

### Buprenorphine supply disruption

Shortages in buprenorphine medication supplies were reported by 38 (43%) participants; however written or telephone refills were eventually obtained from either the clinic providers or an unaffiliated buprenorphine provider (71%), friends (24%), and/or drug dealers (8%) (Table 2). Of note, respondents who obtained buprenorphine from drug dealers reported no concomitant illicit opioid or other drug use, and no increase in the street value of buprenorphine. Reducing total daily doses of buprenorphine, or “stretching,” was reported by 26 participants (29%). Of the patients reducing their total daily doses of buprenorphine, 2 participants had increased or new-onset illicit opioid use. Seven (8%) participants reported having extra quantities of buprenorphine prior to the hurricane.

**Table 2:** Coping strategies among participants that reported buprenorphine supply disruption, n = 38

**Coping strategy**^**a**^	**n (% of 38)**
Obtained an unscheduled buprenorphine refill from a program physician	27 (71)
Obtained buprenorphine from a friend	10 (26)
Tolerated withdrawal symptoms	9 (24)
Obtained buprenorphine from a drug dealer	5 (13)
Used heroin	4 (11)
Used prescription opioids	2 (5)
Obtained buprenorphine from family	1 (3)

^a^patients may have chosen more than option.

Among those with difficulty obtaining refills, five participants reported Medicaid and other insurance prior authorization problems (6%), which were well-known barriers to routine refills prior to the event. Respondents unable to reach clinic buprenorphine providers were unable to obtain guest doses of buprenorphine from emergency rooms (3%) or pharmacies (3%). Some respondents (12%) reported significant crowding and long wait-times at the alternate temporary clinic location, which was providing care in a facility not usually used for clinical care.

Contact with BHC buprenorphine providers on an unscheduled basis or surrounding a previously scheduled appointment appeared to help patients cope with hurricane-associated difficulties. Nearly half of the respondents were called by a BHC primary care clinic buprenorphine provider or BHC employee following Sandy (49%). Most respondents had attempted calling BHC following the hurricane (64%), however only 19 (33%) participants reported having their needs addressed through the general information/operator number.

Respondents were also asked about suggestions to improve clinical services in the event of a disaster or unexpected disruption in services. The most common suggestion was to improve physician-patient communication (36%). Specific suggestions included: providing disaster hotlines, access to buprenorphine providers’ mobile phone numbers and emails, and a frequently updated website with disaster-related information. Other suggestions included improving access to buprenorphine refills (12%) and providing additional supplies of buprenorphine prior to the hurricane (2%).

### Other substance use

**Figure 1:**
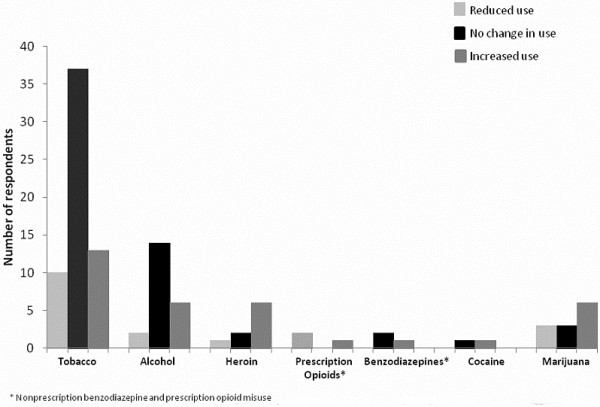
Changes in self-reported substance use between the three months preceding and one month post-disaster, n = 89.

Changes in self-reported tobacco, alcohol, and other drug use during the three months prior to Hurricane Sandy compared to the one-month period following Sandy are displayed in Figure 1. Of importance, marijuana use increased in six of the twelve participants (50%) self-reporting misuse. Among respondents self-reporting cigarette use, 62% reported no changes in the amount of cigarette use following the hurricane while 22% reported increased use post-disaster. A similar proportion of respondents self-reporting alcohol use during the three-months prior to Sandy and one month post-disaster reported no changes in alcohol use (64%); and 27% reported increased alcohol consumption. There was no increased cocaine misuse following the hurricane in both participants reporting use. Among five participants (6%) self-reporting benzodiazepine use prior to Sandy, two continued using the same amount post-disaster and three decreased their quantity.

### Other disaster-related outcomes

Hurricane-related adverse material consequences were reported by 38 (43%) participants. These problems included financial difficulties (27%), prolonged power outages (24%), and loss of housing (9%). Of the 38 respondents reporting hurricane-related adverse effects, assistance was provided by family (34%), friends (26%), relief organizations including FEMA and Red Cross (11%), and peers from 12-step groups (3%). Six (16%) participants reported no receipt of any disaster relief. Neighbors and religious organizations were specifically not reported as sources of help by any participants. Respondents that received assistance were asked to elaborate on the type of help they received, and most commonly reported receiving temporary housing (9%), financial support (8%), food (5%), medical assistance (1%), household supplies (1%), and “other” (3%). Of the participants reporting receiving financial support, help was provided by FEMA (29%), personal savings (29%), illicit income (21%), family and friends (14%), employers (7%). Two participants refused to disclose their sources of financial support. Only two of the 48 respondents reported being paid by employers for lost wages (4%). Most participants resided in their primary residence during the night of the hurricane (78%). Four respondents were not in New York City at the time of the hurricane. Of the eight respondents requiring temporary housing due to storm damage or flooding, one participant reported having to leave one ‘three-quarter’ house for another. There was open drug use at the alternate housing location, to which he attributed his post-event heroin use.

## Discussion

This cross-sectional survey among NYC public sector primary care office-based buprenorphine patients following Hurricane Sandy indicated low rates of new-onset or increased illicit opioid use, and high rates of buprenorphine treatment adherence despite the temporary disruption of regular follow-up visits due to hospital closure. New onset or increased heroin and prescription opioid misuse was rarely reported, despite many patients experiencing buprenorphine medication supply disruptions and lowered daily buprenorphine doses. These findings suggest office-based buprenorphine, in which patients are typically seen for office visits and medication refills weekly-to-monthly while maintaining a medication supply at home, is relatively adaptable to service disruptions following a natural disaster.

### Opioid and other substance misuse

The minimal increase in new onset or increased illicit opioid use among surveyed participants is in line with Weiss’s findings that only one in nine respondents enrolled in a methadone treatment program reported heroin use after the September 11^th^ attacks [11]. However, this contrasts with a larger sample study of methadone patients that found a 34% increase in relapse to heroin [16]. A home supply of buprenorphine may allow patients to cope more effectively in the event of service disruption compared to methadone, which requires frequent clinic visits for directly observed dosing. Patients that encounter a reduced supply of buprenorphine and are unable to obtain refills used an array of strategies to obtain additional doses. Other strategies included obtaining additional doses of buprenorphine from friends, family, and drug dealers.

### Buprenorphine supply disruption

Our findings revealed that close to half of patients experienced shortages in buprenorphine supply following the hurricane. Most patients reported being able to relatively quickly obtain refills from their usual BHC buprenorphine providers, most often by phone followed by mailed written prescriptions to area pharmacies. Temporarily relocating to an affiliate public hospital provided a physical back-up to phone-based refills and provided medication dispensing to uninsured patients. The communication link between patients and providers was an important factor in facilitating call-in refills to local pharmacies (with mailing of original prescriptions), obtaining prior authorizations, and arranging eventual follow-up visits. As shown in Table 1, a higher mean-time in treatment may have led to more robust coping strategies in response to supply disruption; a slightly higher median buprenorphine dose may have permitted greater flexibility in self-directed dose adjustments (“stretching”) post-disaster. In addition, prior studies have suggested that kappa-receptor antagonist action by buprenorphine-naloxone may be associated with reduced dysphoria, mood symptoms, and craving following episodes of prolonged abstinence [17].

Downward adjustments to daily buprenorphine doses and obtaining additional supplies from friends or family was common following Sandy. A quarter of the participants in this study reporting supply disruption were able to obtain extra doses of buprenorphine from friends or family also receiving buprenorphine treatment. An additional third of surveyed patients reported reducing (“stretching”) their daily doses of buprenorphine. While some were forced to do so as a result of reduced supplies of buprenorphine, others proactively rationed their daily use in case of unexpected barriers to accessing refills. Simply defining such behaviors as “diversion” or “treatment non-adherence” fails to acknowledge patients’ earnest attempts to avoid illicit opioid misuse. A home supply of buprenorphine may offer a more robust source of support for patients, as well as their peers, that encounter disasters and disruptions. Policies permitting additional doses of buprenorphine prior to an anticipated disaster event or service disruption may prevent adverse events such as relapse.

### Contacting or being contacted by providers

When asked how the clinic could improve services in the event of a future disaster, the most common response was to improve communication between patients and buprenorphine providers. This feedback was echoed throughout the survey. Patients that had a buprenorphine provider’s cell phone number highlighted how helpful this was throughout the post-disaster period. This was important for the treatment team to acknowledge, as it highlighted the critical role direct contact with primary care based buprenorphine providers had in the immediate disaster mitigation and more extended disaster recovery phases, as opposed to general hospital contact numbers. When asked about coping strategies in the post-disaster period, the starting point for patients requiring refills was not a local emergency room or disaster relief medical station, but their primary care buprenorphine provider.

Prior disaster studies emphasized the importance of improving the continuity of care for those burdened with chronic medical and psychiatric conditions who are at risk of decompensating due to disruptions in transportation and reduced access to healthcare services [18-21]. The relative adaptability of a public sector, office-based buprenorphine clinic as well as respondents’ ability to secure additional supplies of buprenorphine ensured continuity of treatment despite limited preparation prior to the hurricane.

### Study limitations

Limitations to this analysis of self-reported survey data include response and recall biases. In general, our clinic has approximately a 13% baseline rate of inaccurate opioid misuse self-report compared to urine toxicology results (i.e. 13% of visits involve divergent self-report and urine toxicology results) and avoidance of opioid misuse and buprenorphine diversion are overall goals of treatment [3]. Most interviews were conducted by staff known to patients as part of our practice, potentially leading some respondents to under-report substance misuse or other aberrant behavior despite being guaranteed confidentiality. Nonetheless, most were quite forthcoming in describing many types of coping strategies, illicit and licit. Recall bias surrounding retrospective report of health and drug use behaviors pre/post event and a delay between the event and the administration of the survey no doubt limited the analysis. Our initiation of the survey as soon as possible post-event (approximately one month post) was intended to minimize recall bias. Lastly, as our response rate was not 100%, it is possible that the experiences of respondents were not representative of the broader clinic population. However, review of records of non-respondents revealed that most were actually doing very well in care, with low visit frequency due to longstanding abstinence, medication adherence, and psychosocial stability – making it unlikely that non-respondents had significantly greater problems with treatment disruption or substance misuse.

## Conclusion

Further investigation is needed into developing primary care based disaster preparedness and mitigation efforts for patients enrolled in a buprenorphine primary care program, particularly strategies that will strengthen physician-patient communication following service disruption. Additional studies are also needed to determine outcomes among opioid dependent patients enrolled in MTP, private practice buprenorphine treatment, and medication-free treatment. Our findings highlight the relative adaptability of a prescribed home supply of buprenorphine for opioid dependent patients encountering a disaster and temporary clinic closure. We found only minimal increases in self-reported substance use. Offering patients with a back-up location to provide refills, medical, and psychiatric treatment ensured minimal disruption to continuity of care. Ensuring a working phone hotline and immediate post-disaster telephone communication with patients may assist in providing an information clearinghouse and referral system for those requiring the most immediate assistance.

## Competing interests

Drs. Rotrosen and Lee are lead investigators for a multi-site NIDA-funded study that is receiving free study drug (Suboxone film) from Reckitt-Benckiser.

## Authors’ contributions

BT, EG, JR, JDL made substantial contributions to conception and design. EG, BT, RB, ARW have made substantial contributions acquisition of data, BT, JDL, and EG contributed to the analysis and interpretation of data; BT, ARW, RB, JR, EG, JDL were involved in drafting the manuscript. All authors read and approved the final manuscript.
